# Proteomic analysis of protein purified derivative of *Mycobacterium bovis*

**DOI:** 10.1186/s12967-017-1172-1

**Published:** 2017-04-03

**Authors:** Sante Roperto, Mariaconcetta Varano, Valeria Russo, Roberta Lucà, Monica Cagiola, Marco Gaspari, Dora Maria Ceccarelli, Giovanni Cuda, Franco Roperto

**Affiliations:** 1 Dipartimento di Medicina Veterinaria e delle Produzioni Animali, Università di Napoli Federico II, Naples, Italy; 2 Dipartimento di Medicina Sperimentale e Clinica, Università di Catanzaro “Magna Græcia” Campus “S. Venuta”, Catanzaro, Italy; 3 Istituto Zooprofilattico dell’Umbria e delle Marche, Perugia, Italy; 4 Dipartimento di Biologia, Università di Napoli Federico II, Naples, Italy

## Abstract

**Background:**

Tuberculin skin test based on in vivo intradermal inoculation of purified protein derivative from *Mycobacterium bovis* (bPPD) is the diagnostic test for the control and surveillance of bovine tuberculosis (bTB).

**Methods:**

Proteomic analysis was performed on different bPPD preparations from *M. bovis*, strain AN5. Proteins were precipitated from bPPD solutions by TCA precipitation. The proteome of bPPD preparations was investigated by bottom-up proteomics, which consisted in protein digestion and nano-LC–MS/MS analysis. Mass spectrometry analysis was performed on a Q-exactive hybrid quadrupole-Orbitrap mass spectrometer coupled online to an Easy nano-LC1000 system.

**Results:**

Three hundred and fifty-six proteins were identified and quantified by at least 2 peptides (99% confidence *per* peptide). One hundred and ninety-eight proteins, which had not been previously described, were detected; furthermore, the proteomic profile shared 80 proteins with previous proteomes from bPPDs from the United Kingdom and Brazil and 139 protein components from bPPD from Korea. Locus name of *M. bovis* (Mb) with orthologs from *M. tuberculosis* H37Rv, comparative gene and protein length, molecular mass, functional categories, gene name and function of each protein were reported. Ninety-two T cell mycobacterial antigens responsible for delayed-type hypersensitivity were detected, fifty-two of which were not previously reported in any bPPD proteome. Data are available via ProteomeXchange with identifier PXD005920.

**Conclusions:**

This study represents the highest proteome coverage of bPPD preparations to date. Since proteins perform cellular functions essential to health and/or disease, obtaining knowledge of their presence and variance is of great importance in understanding disease states and for advancing translational studies. Therefore, to better understand *Mycobacterium tuberculosis* complex biology during infection, survival, and persistence, the reproducible evaluation of the proteins that catalyze and control these processes is critically important. More active and more specific tuberculins would be desirable. Indeed, many antigens contained within bPPD are currently responsible for the cross-reactivity resulting in false-positive results as they are shared between non-tuberculous and tuberculous mycobacteria.

**Electronic supplementary material:**

The online version of this article (doi:10.1186/s12967-017-1172-1) contains supplementary material, which is available to authorized users.

## Background

Tuberculosis (TB), a zoonotic disease, is a major global human health problem, with 10.4 million new cases of active disease and nearly 1.8 million deaths estimated for 2015 [1]. The disease has similarly heavy consequences for a broad range of animal species thus being a recognized public veterinary health problem in many countries [2, 3].

Tuberculosis in bovines (bTB), caused predominantly by *Mycobacterium bovis* (*M. bovis*) a member of the *Mycobacterium tuberculosis* complex, is a disease still endemic in many countries [4]. bTB is the cause of significant economic hardship to the livestock industry with estimates of >50 million cattle infected worldwide [5] and is of zoonotic importance [6]. Indeed, although *M. bovis* is known to be the most important infectious agent responsible for bTB, however, it may cause tuberculosis in humans (hTB) both in developing and developed countries [7–9].

Moreover, bTB is subject to comprehensive control measures in order to limit both zoonotic transmission and economic losses. Such control is typically based on test-and-slaughter schemes, which require the accurate diagnosis of infected animals [10].

The diagnostic test for the control and surveillance of bTB used worldwide is the Tuberculin Skin Test (TST), which is based on in vivo intradermal inoculation of purified protein derivative from *M. bovis* (bPPD) alone or in combination with *M. avium* (aPPD). Those animals that react to PPD are isolated and slaughtered [11, 12]. Despite intensive eradication efforts over decades, bTB persists as a costly disease with adverse impacts on animal health and welfare, trade of animals and animal products, and livelihoods of producers, and continues to be a problem with global perspectives [4, 13]. It has been suggested that TST is a good herd test but a poor test for identifying individual infected animals [4]. Furthermore limitations in specificity and sensitivity of bPPD are additional factors contributing to the persistence of bTB [14]. However, TST is the gold standard for determining whether an individual animal is infected with bTB.

bPPD is a poorly characterized and ill-defined mix of proteins, lipids, and carbohydrates [11, 13] and little is known regarding what compounds are responsible for the delayed-type hypersensitivity (DTH) response [15, 16]. More defined knowledge on PPD composition and contribution of individual antigens in TST would give a better insight into the molecular mechanism behind the complex would therefore allow a better selection of proteins specific to *M. tuberculosis* [17]. Therefore, the identification of the molecular composition would facilitate the development of a more refined reagent [15].

A few proteomic studies have been performed on bPPD composition. Borsuk et al. [18] reported the first proteomic study from bPPD from the United Kingdom (UK) and from Brazil (BR). Cho et al. [19] described proteome profiles of bPPD from Korea (KR). More recently, Gcebe et al. [20] carried out a proteomic analysis of bPPD obtained from Prionics at The Netherlands.

The aim of the present paper is to report proteomic profiles detected on four bPPD preparations used for control and surveillance of bTB in most European countries.

## Methods

### bPPD samples

Four bPPD preparations from *M. bovis*, strain AN5, were examined. They were from Spain (S), manufactured by CZV company [CZ Veterinaria S.A., Porriño, (Pontevedra) Spain] and commercialized in Spain, Portugal, France, Germany, Ireland, Greece, United Kingdom, Belgium, Hungary, Bulgaria, Italy, Romania; two from Italy (one prepared by Istituto Zooprofilattico dell’Umbria e delle Marche, Perugia (I_p_) and one by Istituto Zooprofilattico Sperimentale dell’Abruzzo e del Molise “G. Caporale”, Teramo (I_t_), respectively) and commercialized in Italy and one from Netherlands (NL), manufactured by Prionics Lelystad BV, Lelystad, Holland and commercialized in Germany, Denmark, Sweden, Norway and Netherlands.

### Chemicals

All chemicals were from Sigma (St. Louis, MO), unless otherwise specified.

### Protein digestion and peptide dimethylation labelling

Proteins were precipitated from bPPD solutions by TCA precipitation. Briefly, proteins were precipitated in 10% TCA overnight, then pelleted at 12,000 g for 30 min at 4 °C. Pellets were washed with (1) ethyl ether (2) acetone. Proteins were resuspended in 100 mM triethyl ammonium bicarbonate buffer (TEAB) containing 0.2% SDS. Protein content was determined by the BCA protein assay using BSA as standard for the calibration curve (BCA protein assay, Thermo Scientific, Rockford, USA). For each sample, a 100 μg aliquot was subjected to protein reduction (10 mM DTT, 1 h at 37 °C) and alkylation (24 mM iodoacetamide, 1 h at 37 °C). The excess of iodoacetamide was quenched by additional 2 mM DTT. SDS concentration was brought to 0.05% by addition of HPLC water; then, 1 μg of proteomics grade trypsin was added, and digestion was allowed to proceed overnight at 37 °C.

Samples were labelled by either “light” (L), “medium” (M) or “heavy” (H) dimethyl labelling [21]. An aliquot of each sample, containing 25 μg of protein, was transferred to a separate Eppendorf vial. Then, 4 μL of either 4% (v/v) regular formaldehyde (L) or CD_2_O (M), or ^13^CD_2_O (H) *plus* 4 μL of either 0.6 M NaBH_3_CN (L, M) or NaBD_3_CN (H) were added. Reductive amination was allowed to proceed at room temperature for 1 h with shaking. To quench the reaction, 16 μL of 1% (v/v) ammonia solution and 8 μL of 5% formic acid were added to the samples. Samples were labelled in duplicates with label swapping, as follows: sample S (L, M, H), sample I_t_ (M, L), sample NL (H, L), sample I_p_ (H, M). Triplets were obtained by combining 5 μg of each labelled sample as follows: (1) S(L):I_t_(M):NL(H); (2) I_t_(L):S(M):I_p_(H); (3) NL(L):I_p_(M):S(H). Sample S was used as reference sample: thus, it was present in all triplets. Each mix was fractionated by SCX StageTips [22] using Empore SCX Extraction Disks. Briefly, samples were diluted 20-fold in solution A (0.5% formic acid, FA in 80% ACN), and then loaded onto a column prepared from a 10 μL micropipette tip stacked with two layers of the SCX resin, previously conditioned with 20 µl of solution A. The plugs were washed twice with 20 μL of solution A. Then, peptides were stepwise eluted by delivering six 14 μL aliquots of eluent of increasing ionic strength. The first five eluent solutions contained 20% acetonitrile and 0.5% formic acid (v/v) plus the following amount of ammonium acetate: (1) 50 mM, (2) 75 mM, (3) 100 mM, (4) 150 mM, (5) 250 mM. The sixth eluent solution was composed of 20% acetonitrile and 500 mM ammonium acetate. Eluates were evaporated to dryness and resuspended in 12 μL of mobile phase A (see below).

### Nano-LC–MS/MS and data analysis

Mass spectrometry analysis was performed on a Q-Exactive Hybrid Quadrupole-Orbitrap Mass Spectrometer coupled online to an Easy nano-LC1000 system (Thermo Fisher Scientific, Germany). Peptide mixtures (3 µL) were loaded at a flow rate of 500 nL/min onto a silica capillary tip (75 µm i.d., length 10 cm) packed in house with 3 µm C_18_ silica particles (Dr. Maisch, Germany). Gradient elution was from 8% B (0,1% FA in 80% ACN) to 35% in 55 min, then from 35% B to 100% B in 5 min. Column equilibration (20 min) was at 2% B. MS acquisition was performed in positive ion mode with a nanoelectrospray voltage of 1800 V. Mass spectra were obtained in data-dependent acquisition (DDA) mode, using a top 12 method consisting in a survey full scan across the *m/z* range 350–1800, followed by MS/MS scans on the twelve most intense precursor ions accumulated for a maximum of 60 ms. Full scan acquisition parameters were: 70,000 FWHM, AGC target 1e6, maximum IT 50 ms, scan range 350 to 1800 *m/z*. Instead, dd-MS^2^ acquisition parameters were set as following: 17 500 FWHM, AGC target 1e5, maximum IT 60 ms, isolation window 1.6 *m/z*, scan range 200 to 2000 *m/z*. Collision energy was set at 25%.

Raw data were processed by Proteome Discoverer 1.4 using the Sequest algorithm and searched against the protein sequence database of *Mycobacterium tuberculosis* Complex (June 2015, 6415 proteins). Search criteria were set as follows: enzyme trypsin, maximum two missed cleavages, Dimethyl (Any N-Terminus), Dimethyl (K), Carbamidomethyl (C) as static modifications (dimethyl modifications were set to either “light”, “medium” or “heavy” in three parallel searches), Oxidation (Met) as dynamic modification, MS tolerance 10 ppm, MS/MS tolerance 0.02 Da. Search results were filtered by q values using Percolator [23], integrated in Proteome Discoverer (q value <0.01). As negative examples for the classifier, Percolator peptide hits derived from searching a decoy database composed of reversed protein sequences were used. Protein hits based on two successful peptide identifications in at least 2 out 3 LC-MS/MS data sets were considered valid. Initial quantification was performed in Proteome discoverer using default parameters for triplex dimethyl labelling. Advanced quantification, such as data normalization and permutation statistical analysis of the peptide ratios was performed using quantitative proteomics p value calculator (QPPC) [24]. Number of permutations was set to 10,000. Minimum number of observations was 2; thus, proteins identified by two peptides but quantified by a single unique peptide were not assigned a fold change. For each binary comparison (I_t_ versus S, NL versus S, I_p_ versus S) proteins whose fold change was either >2.0 or <0.5, with an associated p value <0.05 in both replicate analysis were considered differentially abundant. Finally, BoviList and TubercuList databases (http://tuberculist.epfl.ch/) provided information on annotated *M. bovis* and *M. tuberculosis H37Rv* genes and proteins, including molecular weights and functional annotation as well as orthologous genes of these two highly related strains. Uniprot (http://www.uniprot.org/) and KEGG (http://www.kegg.jp/kegg/) databases have also been utilized in order to obtain further detailed information about proteins of *M. bovis, strain AN5* and *M. tuberculosis, strain H37Rv.*

The mass spectrometry proteomics data have been deposited to the ProteomeXchange Consortium via the PRIDE [25] partner repository with the dataset identifier PXD005920.

## Results

### Identification of bPPD proteins by list-based nano-LC-MS/MS

**Fig. 1:**
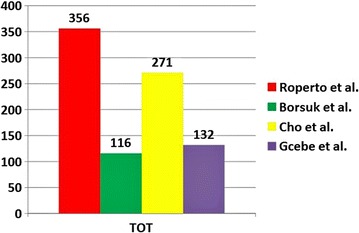
Total number of proteins identified within four bPPD preparations

**Fig. 2:**
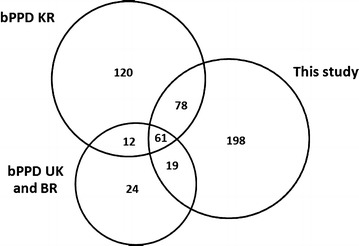
Venn diagram showing the unique and shared proteins detected in this study compared with previous bPPD proteomic profiles

The proteome of four bPPD preparations was investigated by bottom-up proteomics, which consisted in protein digestion and nano-LC-MS/MS analysis. Altogether, we identified 356 proteins by 2 or more peptides (99% confidence *per* peptide) in at least 2 out 3 LC-MS/MS data sets (Fig. 1). A vast majority (~75%) of these proteins was found to be shared among four bPPDs. Our proteomic profile was composed of 198 not previously described proteins; furthermore, nineteen proteins were found to be shared with bPPD UK and bPPD BR proteome [18] and seventy-eight with bPPD KR [19]. Sixty one proteins were found in common both with bPPD UK as well as BR and bPPD KR proteomes (Fig. 2). Additional file 1: Table S1 reports locus names of *M. bovis* (Mb) and their orthologs from *M. tuberculosis* H37Rv (Rv) including comparative gene and protein lengths, molecular mass, functional categories, gene name and protein function of both mycobacterium strains. Finally, Additional file 1: Table S1 shows a comparative protein expression profile with two previous bPPD proteomic analysis [18, 19]. Furthermore, our proteomic profile shared 158 proteins with the proteome of *M. tuberculosis* PPD (MtbPPD) investigated by Prasad et al. [17] who were able to identify 265 proteins, the largest number of proteins found in MtbPPD so far.

### BoviList functional classification codes

The proteins of bPPDs were grouped into eight functional categories, with the most representative (33%) being the functional category 7 (*intermediary metabolism and respiration*). The other proteins were grouped into the functional categories that include 0 (*virulence, detoxification, adaptation*—3%), 2 (*lipid metabolism*—5%), 3 (information pathways—20%), 4 (cell wall and cell processes—13%), 9 (regulatory proteins—4%), 10 (*conserved hypotheticals*—19%) and 16 (*conserved hypotheticals with an orthologue in M. tuberculosis*—2%). From a comparative point of view, all proteins we have identified in bPPDs have protein equivalents from *M. tuberculosis* H37Rv (http://tuberculist.epfl.ch/), but some of them have a different structure and appear to belong to different categories (see Additional file 1: Table S1—red and blue colors, respectively). Accordingly, protein equivalents from *M. tuberculosis* H37Rv were grouped in seven functional categories.

### Mycobacterial proteins containing possible T-cell antigens

Ninety-two mycobacterial proteins containing possible T-cell antigens and responsible for delayed-type hypersensitivity (DTH) have been identified. *M. tuberculosis, strain H37Rv,* and *M. bovis, strain AN5,* are highly related. Accordingly, some proteins including Mb0448, Mb1918c, Mb2002c, Mb3789, Mb3834c, Mb3904, Mb3905, just like their orthologs from *M. tuberculosis H37RV,* could have more than 20 different T cell epitopes since they are known to be evolutionarily hyperconserved [26, 27]. Numerous proteins such as Mb0584, Mb1767, Mb2056c, Mb2057c, Mb2058, Mb 2493c, Mb2656, Mb2657, Mb2659c, Mb3155, Mb3157c, referred to as latency antigens involved in the latent infection, appear to be as a part of the so-called dormancy (DosR) regulon, the expression of which is observed as part of adaptive response of *M. tuberculosis* complex to hypoxia [28, 29].

**Fig. 3:**
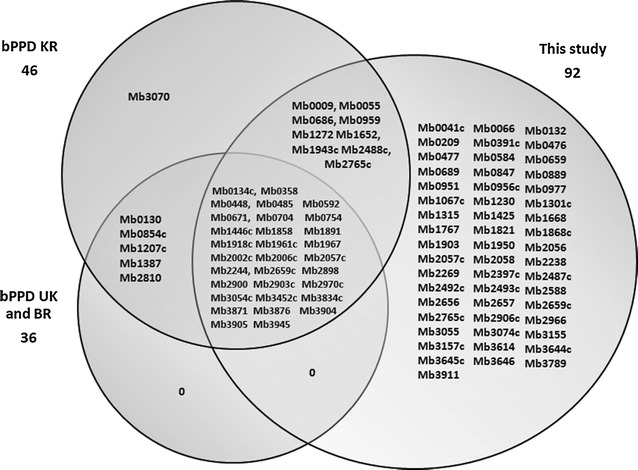
Venn diagram showing the unique and shared T cell mycobacterial antigens seen in this study compared to T cell antigens found in other bPPD proteomic analysis

Fifty-two T cell antigens including Mb1301c (gene name: lprA), Mb1868c (gene name: glcB), Mb1950 (gene name: aceA), Mb3646c (gene name: espA), Mb3911c (gene name: espB) recently identified from *M. tuberculosis,* strain H37RV, via throughput proteome screening [30], have not been reported previously in bPPDs. Fourteen of them belong to a novel series of in vivo expressed *M. tuberculosis* (IVE-TB) T cell antigens [31, 32]. Two reactivation associated antigens, namely Mb0391c (gene name: clpB) and Mb2492c (gene name: rplB) and three lipoprotein T cell antigens, namely Mb0959 (gene name: pstS1), Mb0956c (gene name: pstS2) and Mb0951 (gene name: pstS3), known to generate very high levels of cytokine secretion [33], were also detected. Nine antigens were in common with bPPD KR only [19]. Thirty-one antigens were found to be shared both with bPPDs from UK as well as BR [18] and with bPPD from KR [19]. Our results are summarized in Fig. 3.

### Semi-quantitative analysis of overall protein abundance and quantitative comparison of PPDs by dimethyl labelling

Following database searches, qualitative (Additional file 2: Table S2) and relative quantitative information (Additional file 3: Table S3) was obtained. In Additional file 2: Table S2, proteins are sorted by the parameter “% total weighted spectra”. This is a semi-quantitative measure of protein abundance within a mixture (expressed in estimated % weight), which is based on normalized spectral counting [34]. It is worthwhile noting that the top ten proteins in the list comprise two proteins, namely: ESAT-6-like protein EsxN and Immunogenic protein MPB63, not previously reported. These ten most abundant proteins, whose list is reported in Table 1, constituted over 67% of total weighted spectra.

**Table 1:** The most abundant proteins in PPD preparations

Accession	Description	% Total weighted spectra	I_t_ vs S	NL vs S	I_p_ vs S
61223750	ESAT-6-like protein EsxB	35.0	***0.4*** *0.5; 0.4*	***0.8*** *0.7; 0.9*	***0.5*** *0.4; 0.6*
61223746	6 kDa early secretory antigenic target	15.2	***0.9*** *0.7; 1.0*	***0.5*** *0.3; 0.7*	***0.8*** *0.6; 1.1*
61228252	Immunogenic protein MPB70	5.0	***0.9*** *0.7; 1.1*	***0.6*** *0.5; 0.7*	***0.9*** *0.5; 1.6*
61223754	ESAT-6-like protein EsxN	3.3	***0.9*** *0.6; 1.3*	***0.6*** *0.4; 0.9*	***0.6*** *0.3; 1.0*
61217075	14 kDa antigen	2.2	***1.2*** *1.0; 1.3*	***1.3*** *1.2; 1.4*	***0.4*** ^a^ *0.4; 0.4*
61217901	Meromycolate extension acyl carrier protein	1.9	***0.4*** *0.5; 0.4*	***0.5*** *0.5; 0.6*	***1.0*** *0.9; 1.0*
38605709	10 kDa chaperonin; GroES protein	1.9	***0.3*** *0.3; 0.3*	***0.3*** ^a^ *0.3; 0.3*	***1.5*** *1.2; 1.9*
61228239	Immunogenic protein MPB63	1.5	***0.9*** *1.0; 0.8*	***1.0*** *0.8; 1.4*	***1.0*** *0.9; 1.2*
61233386	50S ribosomal protein L7/L12	1.2	***0.9*** *1.3; 0.6*	***1.0*** *1.2; 0.9*	***0.8*** *0.7; 0.8*
61221045	60 kDa chaperonin 2	1.2	***0.5*** *0.6; 0.4*	***0.5*** *0.6; 0.5*	***0.8*** *0.7; 0.8*

Ten most abundant proteins in PPD preparations; last three columns indicate, respectively, fold change values for samples I_t_, NL and I_p_ with respect to reference S; average fold change is reported in bold italics, whereas values relative to each duplicate analysis are reported in italics

^a^Statistically significant fold change values

Besides estimating average protein abundance by spectral counting, accurate quantification by mass spectrometry based on isotopic labelling was used to compare protein levels in each mixture. Proteins whose relative abundance changed, in both replicates, significantly (p value <0.05) by twofold or more in samples NL, I_t_, I_p_ with respect to the reference sample S, are listed in Additional file 4: Table S4. Comparative analysis by dimethyl labelling showed that PPD preparations did not generally differ significantly in terms of protein composition, especially for what concerns the most abundant proteins. Two notable exceptions were: (1) protein 14 kDa antigen, whose levels in samples NL and I_t_ were comparable to sample S (fold change close to 1), but was found at 0.4-fold change in sample I_p_, and (2) chaperonin GroES, whose levels were comparable in samples I_p_ and I_t_ relative to sample S (fold change close to 1 for I_p_, fold change = 0.3 for sample I_t_, but not found statistically significant in both replicates), but was found at relative fold change 0.3 in sample NL (Table 1). In total, with respect to reference sample S, 44, 34 and 30 proteins were found at significantly different levels in samples I_t_, NL and I_p_, respectively. These proteins were mostly present at very low levels in all preparations.

## Discussion

Comparative proteomics was performed on four bPPD preparations. Proteomic profile of all bPPDs was composed of 356 proteins thus representing the highest proteome coverage of bPPD preparations to date. We deciphered 198 new, never previously reported proteins in the protein expression profile. Altogether, 512 proteins of *M. bovis* PPDs, strain AN5, have been identified so far. Recently, 132 protein components were also revealed from a commercial bPPD preparation [20]. Unfortunately, this last study did not report the complete list of proteins identified by Mb number; therefore, it was not possible to carry out any comparative studies.

The worldwide used diagnostic test for the control and surveillance of bTB is the TST, based on the detection of cell mediated immunity under the exposure to bPPD, the composition of which is highly complex and remains ill-defined [4, 12].

Since proteins perform cellular functions essential to health and/or disease, obtaining knowledge of their presence and variance is of great importance in understanding disease states and for advancing translational studies [35]. Therefore, to better understand *M. tuberculosis* complex biology during infection, survival, and persistence, the reproducible evaluation of the proteins that catalyze and control these processes is critically important [36].

The emerging field of proteomics has contributed greatly to improving our understanding of the *M. tuberculosis* complex quite recently. Proteomics is currently in transition from pure basic research to medical application [37].

All proteins we detected in bPPDs have human equivalents. All bovine and human proteins were characterized by a very strong similarity and a remarkable identity. Indeed, all of them shared characteristics such as gene and protein length as well as molecular mass and function. However, we showed that some bovine and human proteins encoded by ortholog genes belong to different functional categories. Accordingly, it has been shown that some genes of members of *M. tuberculosis* complex can have functional polymorphisms and encoded proteins responsible for some phenotypic differences between *M. bovis* and the other members of *M. tuberculosis* complex [38].

The proteomic content of the four PPD preparations was characterized by a remarkable presence of chaperone proteins such as HspX, DnaK, GroEs, GroEl. These proteins are known to share a high homology (upwards of 70%) and are conserved amongst most mycobacterial species [18, 19]. They are believed to be the main proteins of the current diagnostic test responsible for high level of false positive responses [15].

A quantitative but not qualitative difference in protein content was seen in our bPPD proteomes. It is worthwhile noting that subtle differences in culture conditions, sterilization methods, protein precipitation methods, peptide fractionation process, trypsin efficiency may result in differences in proteomic profiles of bPPDs [19].

It has been suggested that proteomic analysis of different bPPD preparations could improve current diagnostic tests and gain insights into the immune response seen in TB disease [19].

We detected 92 mycobacterial proteins potentially involved in DTH, thus deciphering 52 new antigens not previously reported in bPPDs. Thirty six proteins were identified as T cell mycobacterial antigens in bPPDs from UK and from BR [18]; bPPD KR proteomes revealed 46 proteins playing a role in cellular immunity [19]. Altogether, ninety-eight mycobacterial antigens which play a central role in DTH have been identified in all examined bPPDs. Furthermore, from a comparative point of view, a very large number of T cell antigens was found to be shared between bPPDs of this study and MtbPPDs [26, 28–30, 32, 39–41].

The number of novel T cell mycobacterial antigens is increasingly detected in bPPD. There is a need to establish a better and more detailed understanding of T-cell biology through comparative investigations to decipher immune mechanisms that control mycobacterial infections which appear to rely heavily on the cellular immune system [27, 42]. Accordingly, more active and more specific tuberculins would be desirable. To date, limited progress has been achieved in this field, mainly because of the ill-defined nature of the antigens present in tuberculins as well as the complexity of PPD production.

We believe that developing more proper and defined antigens will be crucial to increase specificity and sensitivity of PPDs. It is worthwhile remembering that there is the urgency to improve specificity of PPD since as cross-reactive responses to bPPD (false-positive results) may occur as many antigens contained within bPPD are shared between non-tuberculous and tuberculous mycobacterial [4, 11].

## Conclusion

Improvement and/or replacement of classical PPD composition with novel more specific reagents remain demanding [15].

To control TB disease, new strategies are needed to prepare better tools based on identification of novel antigens important for developing diagnostic tests which could be not only more accurate and sensitive but also capable of differentiating infected and uninfected vaccinated animals (“DIVA” tests), thus offering perspectives to introduce potential vaccination within existing eradication programs [43, 44].

Indeed, understanding immunity to *M. bovis* is a continuing challenge and one that is of interest to the fields of human and animal medicine alike [6]. It is important to note that ruminant also have greater similarity to humans than do the often-used mouse model with regard to several aspects of immune physiology, increasing the utility of cattle as a model [45]. Therefore, comparative immunology studies will continue to provide mutual benefit to TB research in both man and animals [46].

Accordingly, the One Health approach, believed to be a critical necessity to address zoonotic diseases [2], is clearly warranted for tuberculosis. The One Health concept is a worldwide strategy for expanding interdisciplinary collaborations and communications in all aspect of health care for humans, animals and the environment which, as far as tuberculosis disease is concerned, can speed the development of new diagnostic tests for humans and livestock thus improving tuberculosis surveillance, control, and eradication programs.

## Additional files

**Additional file 1: Table S1.** This table shows: the list of the identified proteins within all four bPPD preparations, ordered locus names of *M. bovis* (Mb) and *M. tuberculosis*, strain H37Rv, (Rv), functional categories of identified proteins, their molecular mass, gene and protein lengths and gene name and protein function. A comparative protein expression profile with two previous bPPD proteomic analysis is also shown. Differences in functional category among identified proteins are in blue; differences in molecular mass, gene and protein lengths are in red. These findings were from TubercuList, BoviList, Uniprot and KEGG databases (See text for further details).

**Additional file 2: Table S2.** The list of identified proteins in bPPDs. Sequence coverage, number of unique peptides, as well as total weighted spectra as a semi-quantitative measure of protein abundance, are reported.

**Additional file 3: Table S3.** Dimethyl labeling-based quantification of proteins present in samples I_t_, NLand I_p_ relative to sample S. Result of single replicates as well as average H:L and M:L ratios, their associated p-values and number of observations (unique peptides) are reported.

**Additional file 4: Table S4.** Proteins found differentially abundant in samples I_t_, NL and I_p_ compared to sample S. The file contains three data sheets reporting 44, 34 and 30 proteins found at significantly different levels in samples It, NL and Ip, respectively. These proteins were mostly present at very low levels in all preparations. Fold changes >2 and <0.5 are reported in two separate lists.

## Abbreviations

TB: tuberculosis
hTB: tuberculosis in humans
bTB: bovine TB
bPPD: bovine purified protein derivative
aPPD: avium purified protein derivative
bPPD BR: bPPD from Brazil
bPPD UK: bPPD from United Kingdom
bPPD KR: bPPD from Korea
DTH: delayed type hypersensitivity
MtbPPD: *M. tuberculosis* PPD
TST: tuberculin Skin Test
I_p_: Istituto Zooprofilattico dell’Umbria e delle Marche, Perugia
I_t_: Istituto Zooprofilattico Sperimentale dell’Abruzzo e del Molise, Teramo
LC-MS/MS: liquid chromatography tandem mass spectrometry
NL: Netherlands
S: Spain
